# The conventional isoproterenol-induced heart failure model does not consistently mimic the diaphragmatic dysfunction observed in patients

**DOI:** 10.1371/journal.pone.0236923

**Published:** 2020-07-30

**Authors:** Ignacio Cabrera-Aguilera, Bryan Falcones, Alicia Calvo-Fernández, Begoña Benito, Esther Barreiro, Joaquim Gea, Ramon Farré, Isaac Almendros, Núria Farré

**Affiliations:** 1 Unitat de Biofísica i Bioenginyeria, Facultat de Medicina i Ciències de la Salut, Universitat de Barcelona, Barcelona, Spain; 2 Heart Diseases Biomedical Research Group, IMIM (Hospital del Mar Medical Research Institute), Barcelona, Spain; 3 Department of Human Movement Sciences, School of Kinesiology, Faculty of Health Sciences, Universidad de Talca, Talca, Chile; 4 Department of Medicine, Universitat Autònoma de Barcelona, Barcelona, Spain; 5 Heart Failure Unit, Department of Cardiology, Hospital del Mar, Barcelona, Spain; 6 Cardiology Department, Hospital Universitari Vall d’Hebron, Vall d’Hebron Research Institute (VHIR), Barcelona, Spain; 7 CIBER de Enfermedades Cardiovasculares, Madrid, Spain; 8 Respiratory Department, Hospital del Mar and Hospital del Mar Medical Research Institute (IMIM), Barcelona, Spain; 9 Health and Experimental Sciences Department (CEXS), Universitat Pompeu Fabra, Barcelona, Spain; 10 CIBER de Enfermedades Respiratorias, Madrid, Spain; 11 Institut d’Investigacions Biomèdiques August Pi i Sunyer, Barcelona, Spain; Scuola Superiore Sant’Anna, ITALY

## Abstract

Heart failure (HF) impairs diaphragm function. Animal models realistically mimicking HF should feature both the cardiac alterations and the diaphragmatic dysfunction characterizing this disease. The isoproterenol-induced HF model is widely used, but whether it presents diaphragmatic dysfunction is unknown. However, indirect data from research in other fields suggest that isoproterenol could increase diaphragm function. The aim of this study was to test the hypothesis that the widespread rodent model of isoproterenol-induced HF results in increased diaphragmatic contractility. Forty C57BL/6J male mice were randomized into 2 groups: HF and healthy controls. After 30 days of isoproterenol infusion to establish HF, *in vivo* diaphragmatic excursion and *ex vivo* isolated diaphragm contractibility were measured. As compared with healthy controls, mice with isoproterenol-induced HF showed the expected changes in structural and functional echocardiographic parameters and lung edema. isoproterenol-induced HF increased in vivo diaphragm excursion (by ≈30%, p<0.01) and increased by ≈50% both *ex vivo* peak specific force (p<0.05) and tetanic force (p<0.05) at almost all 10–100 Hz frequencies (p<0.05), with reduced fatigue resistance (p<0.01) when compared with healthy controls. Expression of myosin genes encoding the main muscle fiber types revealed that *Myh4* was higher in isoproterenol-induced HF than in healthy controls (p<0.05), suggesting greater distribution of type IIb fibers. These results show that the conventional isoproterenol-induced HF model increases diaphragm contraction, a finding contrary to what is observed in patients with HF. Therefore, this specific model seems limited for translational an integrative HF research, especially when cardio-respiratory interactions are investigated.

## Introduction

Heart failure (HF) is a very prevalent disease and a major public health problem with considerably associated mortality and health system expenditure. Noteworthy, the current high prevalence of HF is expected to increase worldwide as a result of the obesity epidemics and population ageing [1, 2]. In addition to primary cardiocirculatory alterations, HF also impacts the respiratory system by inducing lung edema and breathlessness/dyspnea [3, 4], particularly in patients with advanced stage of the disease and appearing either in stable conditions and during acute HF exacerbations. Remarkably, it has been reported that patients with HF also present weakness of the respiratory muscles, specifically the diaphragm [5], which in association with lung edema could contribute to hinder correct ventilation, thus promoting hypoxia and dyspnea.

Accordingly, animal models for optimally studying the pathophysiology of HF not only must exhibit the cardiac alterations characterizing the disease but also should realistically mimic the diaphragmatic dysfunction observed in patients with HF, particularly when studying exercise interventions in HF animal models [6–8]. The HF model based on infusion of isoproterenol is widely used since it features realistic structural and functional cardiac alterations and has the advantage of being experimentally simple [9]. Indeed, contrary to requiring major surgeries as when HF is induced by myocardial infarction or aortic ligation, HF is induced almost non-invasively by simply placing a subcutaneous pump to continuously infuse isoproterenol. It is noteworthy, however, that there are no data describing whether this widely used HF model results in diaphragm dysfunction. Nevertheless, indirect data from research in other fields where isoproterenol is applied at different doses and modes suggest that this agent elicits an increase in the contractile performance of skeletal muscles [10, 11] and in particular of the diaphragm. For instance, it was observed that intravenous injection of isoproterenol enhanced contractility of canine diaphragm [12]. Moreover, in isolated muscle testing of rats with septic peritonitis, isoproterenol added to organ bath increased diaphragmatic contractility [13], similarly as reported when imposing cardiac pressure overload by transverse aorta constriction in a rodent model [14].

In case that, as indirectly suggested from the afore mentioned studies [12–14], application of systemic isoproterenol with the specific dosage and duration as in the HF model would result in diaphragm reinforcement ―exactly the reverse alteration found in HF patients― the cardio-respiratory interest of this model would be challenged. Therefore, the aim if this study was to test the hypothesis that the widespread rodent model of HF based on isoproterenol infusion results in increased diaphragmatic contractility. To this end, diaphragmatic function in the conventional murine model of isoproterenol-induced HF was assessed *in vivo* by ultrasound echography and *ex vivo* by isolated muscle contractility testing.

## Materials and methods

### Animals

The study was carried out on forty adult male C57BL/6J mice (10 weeks old; Charles River Laboratories, Saint Germain sur L’arbresle, France) maintained on a 12 h light/dark cycle room (light on 8:00 am to 8:00 pm) with water and food *ad libitum*. In a first series, 20 mice were randomly assigned to HF and healthy controls (N = 10 each) and were subjected to non-invasive evaluation of muscle function with echocardiography. Similarly, in a second series of mice, *ex-vivo* diaphragm contraction was measured. Diaphragm samples were used for assessing gene expression of myosin types. The intervention protocols were approved by the Ethics Committee for Animal Experimentation of the University of Barcelona.

### Heart failure model

HF was induced by continuous infusion of isoproterenol with an osmotic pump, following the conventional procedure in this model [9]. Briefly, mice were anesthetized with a mixture of inhaled isoflurane and oxygen-enriched air (1.25% during induction and 1% during maintenance) and a small incision was made on the back of each animal between the shoulder blades after removing the hair from the area using depilatory cream. The skin was carefully separated from underlying connective tissues using blunt-ended scissors and an osmotic mini-pump (Alzet, model 1004) containing isoproterenol (Sigma Aldrich) at 30 mg/kg per day dissolved in sterile 0.9% NaCl solution or only 0.9% NaCl solution (for healthy controls) was implanted subcutaneously for delivery pharmacological agent or placebo for 30 days and the incision was sutured with surgical staples (Autoclip, Fine Science Tools). The procedure was performed under aseptic conditions. The surgery platform was continuously warmed to maintain body temperature until the end of anesthesia. Buprenorphine (0.1 mg/kg) was subcutaneously administered 10 minutes before surgery and after 24 hours. Suture staples were removed 7 days after surgery.

### Assessment of heart failure by echocardiography

At baseline (before pump implantation) and 30 days after treatment with isoproterenol or saline (end-point), echocardiography (Vivid IQ and L8-18i-D Linear Array 5-15MHz, General Electric Healthcare, Horten, Norway) was measured by a single operator (NF) who was blind to the animal group following a standard protocol [15]. Briefly, using the same anesthesia as for mini-pump implantation, chest and abdominal hair were removed using depilatory cream, then the mouse was placed in supine position on a continuously warmed platform to maintain body temperature and the four limbs were fixed. Ultrasound gel was applied on the left hemithorax and the following echocardiographic indices were subsequently computed: left ventricular end-diastolic (LVEDD) and end-systolic diameter (LVESD), left ventricular ejection fraction (LVEF) and fraction shortening (FS).

### *In vivo* diaphragmatic echography

Diaphragmatic echography was performed immediately after echocardiography by the same operator and with the same device following a standard protocol for non-invasively measuring diaphragm function in mice [16]. After applying gel on the area overlying the diaphragm just below the rib cage, an ultrasound probe (Vivid IQ and L8-18i-D Linear Array 5-15MHz, General Electric Healthcare, Horten, Norway) was placed along the transverse mid-sternal axis of the mouse, in order to locate the diaphragm on both sides of the body and M-mode was used to measure the diaphragm movement during normal breathing cycles, detecting contraction (positive deflection) and relaxed state (negative deflection) of diaphragm. Diaphragmatic excursion was quantified as the amplitude of movement between the lowest and peak point of the contraction.

### *Ex vivo* assessment of diaphragmatic contractile function

Contractile function of the diaphragm at end point was assessed *ex vivo* using an isolated muscle test system (Aurora Scientific, Aurora, ON, Canada). All force data were recorded using a customized software implemented in LabVIEW (National Instruments, Austin, TX, USA) at a sampling rate of 1000 Hz and analyzed with MATLAB (The MathWorks, Natick, MA, United States). Diaphragm dissection and preparation of muscle strips were carried out following previously described procedures [17]. Immediately after euthanasia by exsanguination, diaphragms were dissected with ribs attached and placed into ice-cold buffer (118 mmol/L NaCl, 4.7 mmol/L KCl, 2.5 mmol/L CaCl_2_, 1.2 mmol/L KH_2_PO_4_, 0.57 mmol/L MgSO_4_, 25 mmol/ L HEPES and 5.5 mmol/L glucose; pH 7.2). This ringer solution was continuously bubbled with a mixture of 95% O_2_ and 5% CO_2_. Diaphragm strips were prepared with the ribs at the distal end and the central tendon at the proximal end. The side of the rib was anchored and kept fixed, the central tendon was attached to a force transducer (305C Dual-Mode Muscle Lever, Aurora Scientific) using sutures [18] and the diaphragm strip was submerged into the above oxygenated ringer solution at 22°C to prolong muscle stability for testing. The muscle strip remained at rest for 5 minutes prior to functional testing. Supramaximal stimulation conditions and optimum length was determined following established procedures [17].

A single twitch was elicited for 3 times (supra-maximal stimulation, 0.5 milliseconds) from which twitch force (peak force), time to peak force (contraction time) and time to 50% relaxation (half-relaxation time) were determined. The force-frequency relationship was then determined by sequentially stimulating the muscle strips at 10–100 Hz (10 Hz intervals) for 1 second at each stimulus frequency interspersed by 2-minutes recovery intervals between each stimulus, allowing measuring the maximum tetanic force. Fatigue resistance was measured as the decay time in force production while the diaphragm was stimulated continuously at 50 Hz for 40 seconds. From this curve we calculated the time until the initial maximum force decreased to 50% [18] and the strength decrement index (SDI) as a decay of force production at second 30 respect to the maximum force production [19]. Finally, the ribs and central tendon were removed, and the wet mass of the muscle tissue was weighted. Muscle cross-sectional area (CSA) was computed as CSA = m/(l·d), where m and l are strip mass and length, respectively, and d (= 1.06 g/cm^3^) is muscle density [17, 20]. Preparation of diaphragm strips was performed by a researcher (IC-A) who was blind to the mice groups. Indeed, the optimum length of diaphragm strips did not show significant differences when comparing HF (7.74 ± 0.15 mm^2^) and healthy control (7.62 ± 0.33 mm^2^) mice (p = 0.751; t-test). Strip weight was 23.8 ± 1 mg and 23.5 ± 1 mg for HF and healthy controls, respectively, with no difference between groups (p = 0.866; t-test). Consistently, strip CSA showed no significant differences when comparing HF (3.0 ± 0.1 mm^2^) and healthy controls (3.2 ± 0.2 mm^2^) mice (t-test p = 0.604).

### Myosin gene expression in the diaphragm

Diaphragm samples extracted from the animals immediately after sacrifice were snap frozen in liquid nitrogen and stored at -80°C for further analysis. All reagents were purchased from ThermoScientific (Waltham, MO) unless specified. RNA was isolated using the RNeasy kit (Qiagen, Hilden, Germany) following the manufacturer’s instructions. Briefly, after all diaphragm samples were collected, they were thawed and immediately lysed in a Polytron PT2100 homogenizer (Kinematica AG, Lucerne, Switzerland). RNA isolated from the tissue was employed to synthetize cDNA by reverse transcription polymerase chain reaction (PCR; TaqMan Reverse Transcription Reagents). Afterwards, changes in gene expression were analyzed by qPCR using the Taqman Fast Advanced Master Mix in a StepOne Plus thermocycler. The candidate genes analyzed were chosen regarding its association with a specific muscle fiber type. Hence, the muscle fiber type MyHC-I correlates with *Myh6*, MyHC-IIa with *Myh2*, MyHC-IId/x with *Myh1* and MyHC-IIb with *Myh4* [21, 22]. Expression of the four genes was normalized to the expression of peptidylprolyl isomerase A (*PPIA*) used as an internal control. Relative gene expression levels are expressed as fold-change of the 2^-ΔCt^ compared to the baseline group of healthy control mice [23].

### Assessment of lung edema

Lungs obtained immediately after diaphragm excision were stored at -80 °C and subsequently thawed at room temperature for 4 h, dried in an oven at 80 °C for 48 h and weighted again. Edema in each lung was assess as the wet/dry (W/D) weight ratio [24].

### Statistics

All data are presented as mean ± SEM. Comparison of *ex vivo* variables between HF and healthy control groups were carried out by t-tests. When normality tests failed, the Mann-Whitney non-parametric test was used. In the case of *in vivo* variables (echocardiography and diaphragm echography) where data for each animal were available both at base line and end-point, comparisons were carried out for the variable change from baseline to end-point. For all tests, p<0.05 was considered as statically significant.

## Results

As expected from previous reports using the isoproterenol model, mice showed echocardiographic indices characteristic of HF. As a result of random distribution of animals among HF and healthy control groups, baseline cardiac indices (LVEDD = 3.49 ± 0.05 mm, LVESD = 2.27 ± 0.06 mm, LVEF = 71.20 ± 1.29%, FS = 35.20 ± 1.08%) did not show significant differences between groups. However, at end-point mice in the HF group showed a significant increase in structural parameters (LVEDD and LVESD) and a significant decrease in functional parameters (LVEF and FS) as compared with healthy controls, confirming heart hypertrophy and decay in cardiac function (Table 1). As indicated in Table 2, HF mice showed slight but significant increase in body weight as compared with healthy controls (Table 2). Moreover, organ weight showed that the animals in HF group exhibited heart hypertrophy (Table 2). Consistently with HF, mice in the isoproterenol group had pulmonary edema since their lung W/D weight ratio was significantly greater than in healthy controls (Table 2).

**Table 1:** The isoproterenol-induced Heart Failure (HF) model significantly modifies echocardiographically-measured left ventricular structure and function.

	Baseline	Healthy ControlΔ	Heart FailureΔ	p value
**LVESD (mm)**	2.27 ± 0.06	-0.17 ± 0.10	-0.54 ± 0.05	0.008
**LVEDD (mm)**	3.49 ± 0.05	-0.13 ± 0.11	-0.59 ± 0.05	0.019
**LVEF (%)**	71.20 ± 1.29	-0.80 ± 1.75	-8.50 ± 1.37	0.003
**FS (%)**	35.20 ± 1.08	-0.70 ± 1.45	-6.20 ± 0.98	0.006

Δ indicates the change in heart variable from baseline to day 30 after isoproterenol or placebo infusion start for each heart failure and in healthy control animals, respectively (n = 10 each group). LVEED: end-diastolic diameter; LVEF: left ventricular ejection fraction; FS: fraction shortening. Values are mean ± SEM.

**Table 2:** The isoproterenol-induced Heart Failure (HF) model induces cardiac hypertrophy and lung edema.

	Healthy Control	Heart Failure	p value
**Body weight (BW) (g)**	28.71±0.52	30.77±0.61	0.028
**Heart weight (HW) (g)**	0.15±0.004	0.19±0.012	0.0001
**Normalized HW (100·HW/BW)**	0.52±0.01	0.64±0.05	0.011
**Lung edema (W/D)**	4.58±0.19	5.93±0.32	0.004

Data were measured in the HF and heathy control and the groups at end point (after 30 days of continuous perfusion of isoproterenol or placebo, respectively). Lung edema index was measured as the ratio between wet (W) and dry (D) lungs (see Methods for explanation). Values are mean ± SEM.

**Fig 1:**
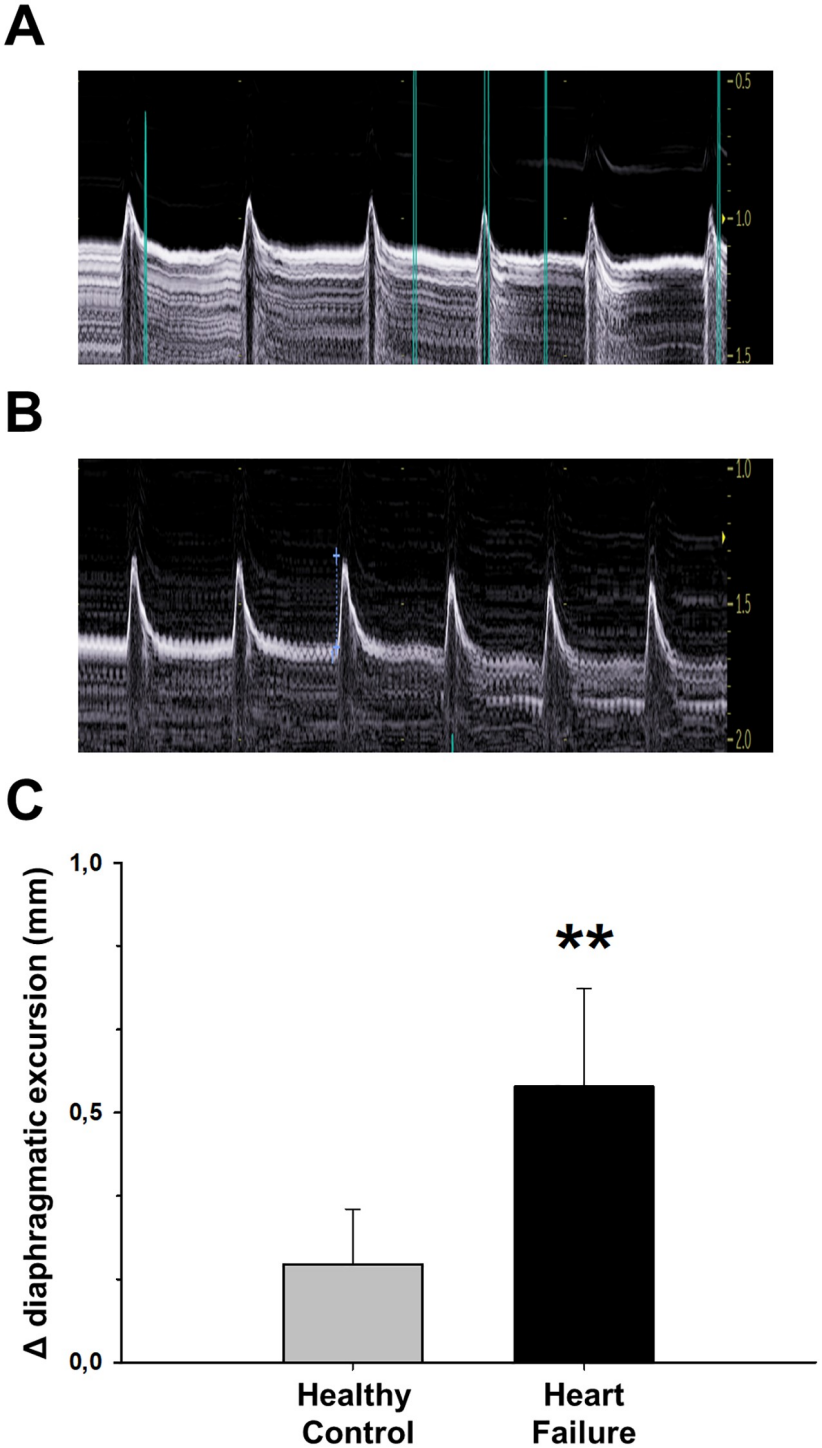
The isoproterenol-induced Heart Failure (HF) model significantly modifies echocardiographically-measured diaphragm function. Diaphragm echography in a representative HF mouse at base line (A) and at end-point (after 30-day of continuous isoproterenol infusion) (B), showing increased excursion during spontaneous breathing. Figures in the excursion scale in the right side of (A) and (B) are mm. (C) Δ indicates the change in diaphragm excursion from baseline to day 30 after starting isoproterenol infusion in HF and in healthy control animals. Values are mean ± SEM. **: *p*<0.01.

In contrast with findings in HF patients, diaphragm function in mice subjected to isoproterenol-induced HF was considerably enhanced as compared with healthy animals, both in *in vivo* and *ex vivo* measurements. *In vivo* echography confirmed that, whereas diaphragmatic excursion at baseline was 2.12 ± 0.10 mm, with no significant differences between groups (p = 0.600), the increase in diaphragm excursion was significantly higher in HF mice as compared with healthy animals at the end of the experiment (Fig 1).

**Fig 2:**
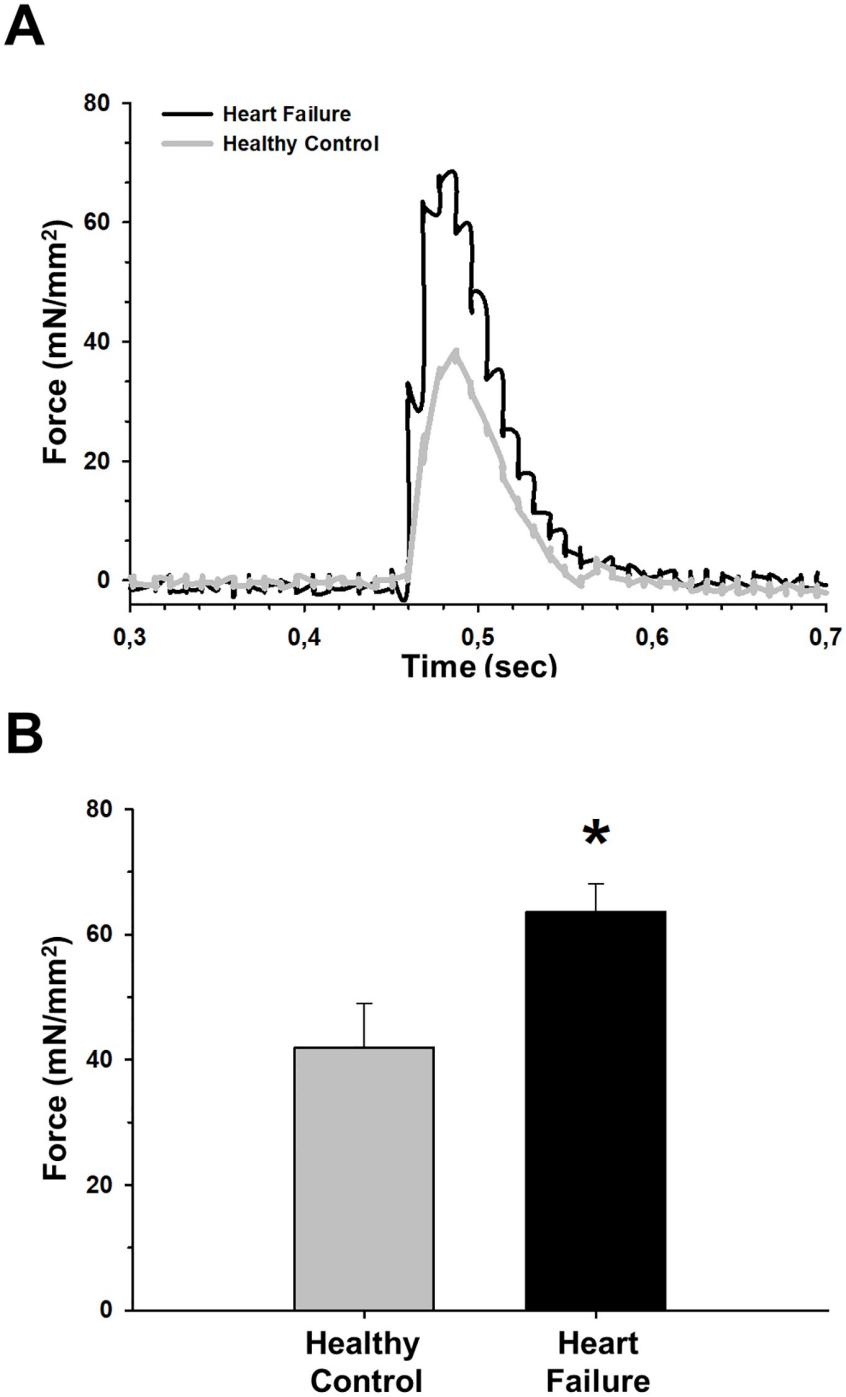
Diaphragm contractile force in a single supramaximal twitch is improved in the isoproterenol-induced heart failure (HF) model. (A) Representative examples of force recordings from healthy (gray) and HF (black) groups. (B) HF animals showed an increase in peak force with respect to healthy mice. Values are mean ± SEM. *: *p*<0.05.

Moreover, *ex vivo* muscle assessment also showed increased diaphragm contractility in isoproterenol-induced HF as compared with healthy controls. Indeed, HF diaphragms exhibited a significant increase in peak specific twitch force in response to a single supra-maximal stimulus compared with healthy controls (Fig 2).

**Fig 3:**
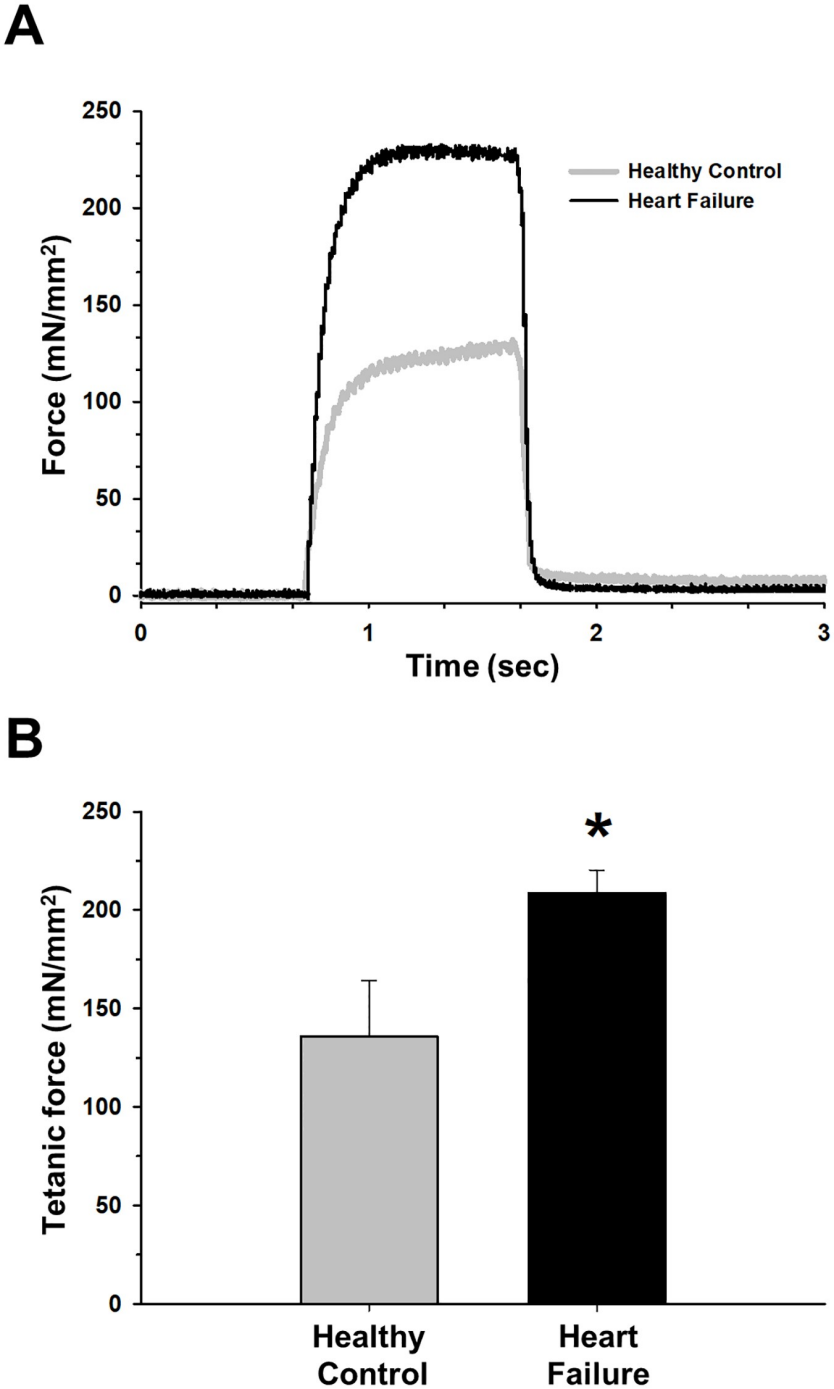
Diaphragm contractile force in a continuous stimulus to generate tetanic contraction is improved in the isoproterenol-induced heart failure (HF) model. (A) Representative examples of tetanic contraction records from healthy (gray) and HF (black) groups. (B) HF animals showed an increase in tetanic force respect healthy group. Values are mean ± SEM. *: *p*<0.05.

However, contraction time corresponding to this stimulus did not significantly (p = 0.397) differ between HF (0.03±0.03 ms) and healthy controls (0.03±0.04 ms). Also, non-significant differences (p = 0.294) were found in half relaxation time (0.03 ± 0.003 ms for healthy controls and 0.04 ± 0.003 ms for HF animals). Maximum force production observed with tetanic contraction using a continuous stimulus was higher in HF animals (Fig 3).

**Fig 4:**
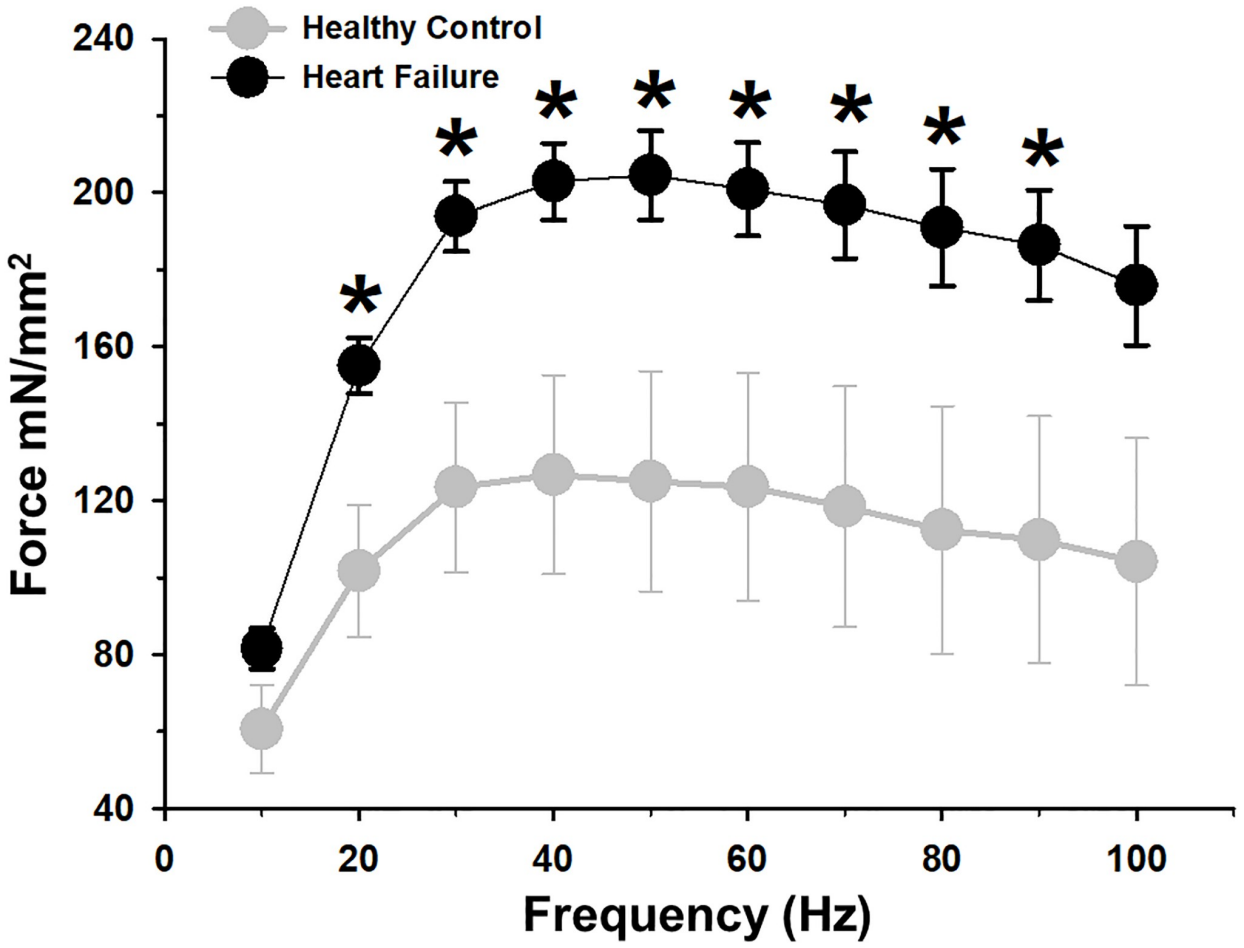
Force frequency relationship between groups at different incremental frequencies. Isoproterenol-induced heart failure (HF) model showed a significant increase in force production for almost all frequencies respect healthy animals. Values are mean ± SEM. *: *p*<0.05.

We also evaluated the tetanic contraction at increasing stimulation frequencies to describe the force-frequency relationship, confirming a significant increase of force production in almost all frequencies in the HF group when compared with healthy mice (Fig 4).

**Fig 5:**
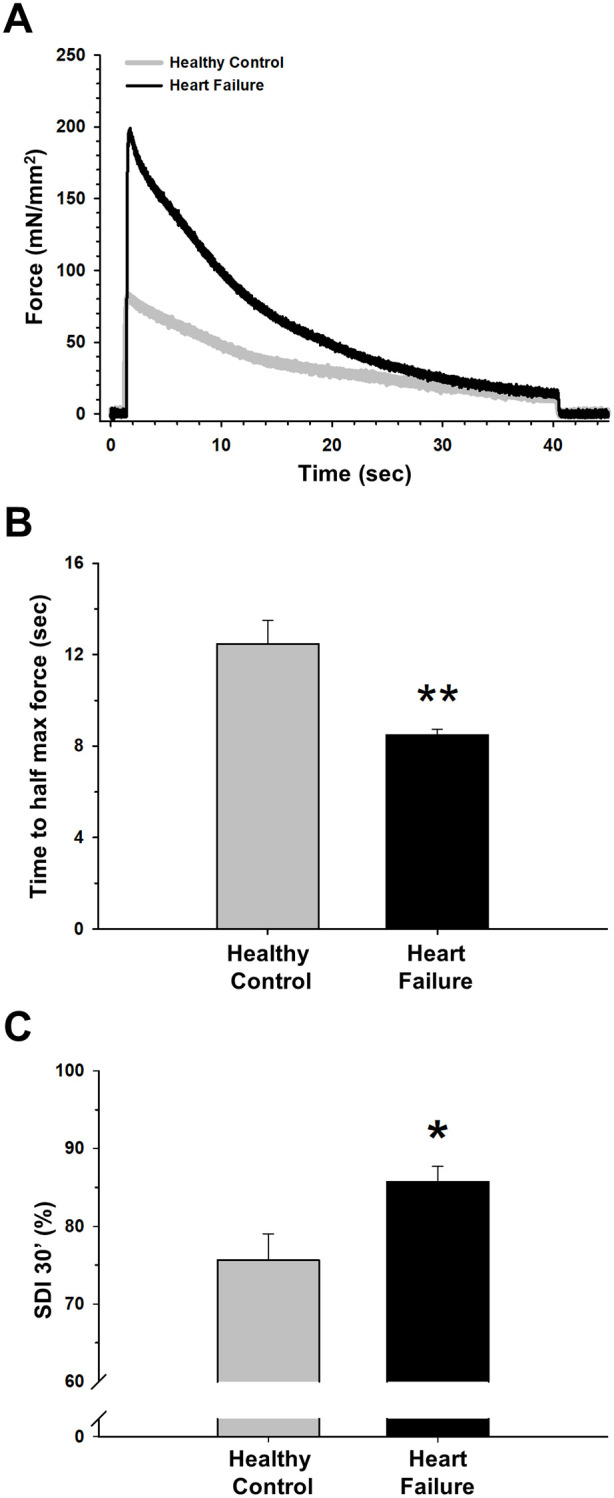
Diaphragmatic fatigue in the isoproterenol-induced Heart Failure (HF) model. (A) Representative examples of fatigue stimulus and decay of force production of healthy and HF diaphragms. (B) HF animals showed a decrease in time to half maximum force and a significant increase in strength decrement index (C) as compared with healthy mice. Values are the mean ± SEM. *: *p*<0.05 and **: *p*<0.01.

Finally, fatigue resistance experiments showed a significant decrease in time to half initial force in HF when compared with healthy control mice and a significant increase in the same group in the strength decrement index (SDI) at 30 seconds (Fig 5), indicating increased fatigue in HF diaphragms.

**Fig 6:**
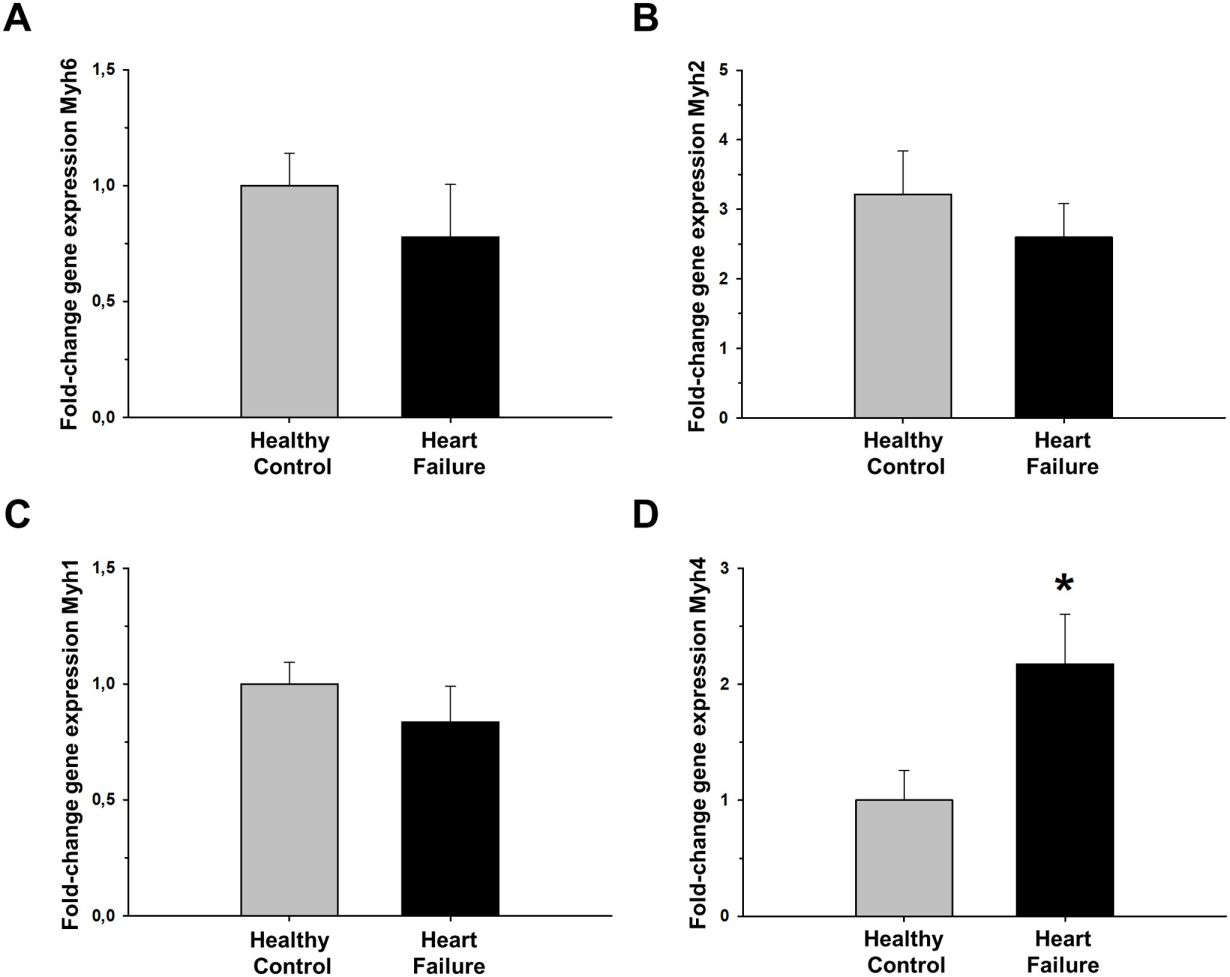
Gene expression of Myh6, Myh2, Myh1 and Myh4 in diaphragm muscle of isoproterenol-induced Heart Failure (HF) mice. (A)Myh6, (B) Myh2, (C) Myh1 and (D) Myh4 as fold-change compared to control healthy mice. Values are the mean ± SEM. *: p<0.05.

Gene expression of *Myh6*, *Myh2*, and *Myh1* at the diaphragm samples showed no significant differences when comparing HF and healthy mice. By contrast, *Myh4* expression showed a significant 2-fold increase in HF animals (Fig 6).

These results support a greater predominance of type IIb fibers over other types of muscle fibers in diaphragm samples of isoproterenol-induced HF animals.

## Discussion

The results of this study reveal that the conventional HF rodent model based on continuous infusion of isoproterenol for 30 days is associated with considerable enhancement of diaphragm contractility. Therefore, whereas this model is very effective in mimicking the cardiac alterations and lung edema characterizing HF, it fails in reproducing the well-known weakening of diaphragm in patients with HF [5], which has been extensively documented by measuring muscle strength in voluntary maximal inspiratory or sniff maneuvers [24–31] as well as in no-volitional measures using phrenic nerve stimulation [25, 29, 32, 33], and which is associated with breathlessness/dyspnea, loss of functional capacity, exercise intolerance, reduced levels of quality of life and survival in HF patients [34–39].

The methodology of the present study for both setting the HF model and for assessing diaphragm contractibility has been widely used in the literature. Indeed, we implemented the mouse model of HF by applying a conventional procedure (subcutaneous pump, dose and duration of isoproterenol application). Accordingly, mice experienced the expected HF structural and functional cardiac changes: increase in LVEDD (by 17%) and in LVESD (by 24%) for structural parameters and decay in functional parameters LVEF (by 12%) and FS (by 18%) as compared with healthy controls. Moreover, HF consistently presented significant lung edema (increase by 29% in the W/D index). Regarding assessment of diaphragm contractibility, we used echography and *ex vivo* specific force measurements. Echography has been validated for detecting time-dependent changes in diaphragmatic function, showing excellent correlation with *ex vivo* force measurement over a wide range of diaphragm excursion values ranging from wild type mice to mutants of Duchenne muscular dystrophy with/out treatment [40]. *Ex vivo* force measurements were carried on diaphragm strips following conventional procedures, achieving values of peak specific twitch and tetanic forces in control mice (≈40 and ≈135 mN/mm^2^, respectively) which were very close to the ones reported for wild type mice when using a similar methodology (≈35 and ≈170 mN/mm2, respectively) [41]. This *ex vivo* experimental model allowed us to focus on muscle tissue contractibility thus avoiding the potential effect of isoproterenol on neural activation.

The increase in diaphragm contractility in the isoproterenol-induced HF in mice, as compared with healthy controls, was considerable either when assessed noninvasively as diaphragm excursion (by ≈30%) or when measured *ex vivo* (by ≈50% in both peak specific twitch and tetanic forces). Such enhancement in diaphragm function in the isoproterenol model strongly contrasts with findings in other rodent models of HF at similar timepoints. For instance, diaphragm dysfunction has been reported in rat models where HF is induced by left coronary artery ligation [7, 8, 42–45], aorto-caval fistula or aortic banding [46–48] or monocrotaline administration [49]. Regarding mouse models, diaphragm weakening has also been reported when HF is induced by left coronary artery ligation [6, 50–52], transverse aortic constriction [16, 53] or in transgenic mice [54]. Therefore, the novel data reported in the present study on the increased diaphragm contractibility observed in isoproterenol-induced HF at 30 days indicates that, as far as this respiratory muscle function is concerned, the model does not behave as in HF patients and in other rodent models of the disease.

Isoproterenol is a nonselective beta-adrenergic agonist inducing early cardiac hypertrophy and hypercontractility followed by HF with cardiac dilation and ventricular dysfunction secondary to by chronic adrenergic overstimulation [9]. The diaphragm contractility enhancement we report here for the first time in the conventional isoproterenol-induced HF model was hypothesized based on previous indirect data on the effects of this nonselective beta-adrenergic agonist in non-cardiac muscles. Indeed, it was known that the consequences of systemic or global administration of isoproterenol can affect skeletal muscles by activating their beta receptors responsible of fiber contraction. In contrast to the morphological and functional changes described in the heart, chronic stimulation of beta receptors have been shown to prevent muscle atrophy in peripheral skeletal muscle in denervated rats [55]. Also, dietary administration of beta adrenoceptor agonist in rats produce growth-promoting protein anabolic effects in muscle tissue [56]. Chronic administration of these agonists causes hypertrophy of skeletal muscle in mice [57] and hypertrophy of diaphragm in hamsters [58]. Moreover, when investigated in different experimental settings other than the HF model, isoproterenol increased canine and rodent diaphragm contractibility [12–14]. It is interesting to note that isoproterenol may also increase the contractile force of skeletal muscles, as it has been reported when added to the bath of *ex vivo* preparations of mouse soleus and extensor digitorum longus [10, 11]. However, whether continuous chronic infusion of isoproterenol as in the HF mouse model induces enhancement of skeletal muscle contractibility is unknown.

Noteworthy, in addition to production of enhanced force, the diaphragm of isoproterenol-induced HF failure mice also showed fast decrease rate and hence a tendency to fatigue (Fig 5). Interestingly, an increase in force production and less fatigue resistance are both characteristic features of muscles with a greater distribution of type IIb fibers [59–61]. The significant increase in *Myh4* (related to MyHC-IIb, the most fast-glycolytic muscle fiber type) in our HF mice (Fig 6) could partially explain the results observed in the *ex vivo* muscle testing. This fiber distribution contrasts with the results reported from diaphragm biopsies in patients with severe HF, showing a shift from fast to slow fibers with higher levels of type I and lower levels of type II fibers compared with healthy controls [62]. Likewise, in animal models of chronic HF that adequately reproduce diaphragmatic weakness some studies also described the same tendency with increase in type I and IIa muscle fibers accompanied by decreases in type IId/x and IIb fibers [43, 47, 63]. The shift from a more fast-glycolytic to a slow-oxidative metabolism in HF partially explains diaphragm weakness and his relationship with breathlessness/dyspnea and exercise intolerance. Therefore, although more detailed fiber analysis could be carried out, our results from myosin gene expression in diaphragms from isoproterenol-induced HF (Fig 6) suggest that changes in fiber types differ from those described in HF patients and explain the greater force production together with the greater vulnerability to fatigue.

This study has some limitations. First, to selectively identify the effect of isoproterenol, a subcutaneous pump releasing saline was implanted into the mice in the healthy control group. It should be noted, however, that implantation of such a subcutaneous pump is a minor procedure with very unlikely systemic consequences. Second, as we mainly focused on documenting the existence of isoproterenol-induced diaphragm increase in contractile force, we did not focus on involved mechanisms, such as whether force production correlated to Ca+2 handling, or on ultrastructural analysis of the diaphragm fibers. the HF model was evaluated at 30 days of continuous isoproterenol infusion, which is a previously validated method [64]. Whether further beta-adrenergic stimulation during longer periods of time could lead to greater vulnerability to diaphragm fatigue and secondary dysfunctional contractility (reproducing the effects of chronic beta-stimulation at the heart) is unknown. However, our preliminary data showing mRNA expression of myosin genes diverging from those described in other HF models with similar timepoints and in HF patients do not support this hypothesis. Interestingly, detailed analysis at different time points in the model would allow characterizing the progression of the diaphragmatic alterations and the correlation between the magnitude of diaphragm contractibility and the severity of heart failure [14].

## Conclusions

In summary, this study has demonstrated a previously unreported and relevant limitation of the conventional rodent model of isoproterenol-induced HF. Indeed, whereas this model is suitable for mimicking the cardiac structural and functional alterations in HF, its considerable increase in diaphragm contractibility is the reverse of the diaphragmatic weakening observed in patients with this disease, thereby questioning its translational interest in HF research, especially when aimed at integrating cardiorespiratory alterations.
